# The Molecular Signature Underlying the Thymic Migration and Maturation of TCRαβ^+^CD4^+^CD8^-^ Thymocytes

**DOI:** 10.1371/journal.pone.0025567

**Published:** 2011-10-13

**Authors:** Fei Teng, Yubin Zhou, Rong Jin, Yu Chen, Xiaoyan Pei, Yuanfeng Liu, Jie Dong, Wei Wang, Xuewen Pang, Xiaoping Qian, Wei-Feng Chen, Yu Zhang, Qing Ge

**Affiliations:** Department of Immunology, Peking University Health Science Center, Beijing, China; Institute of Zoology, Chinese Academy of Sciences, China

## Abstract

**Background:**

After positive selection, the newly generated single positive (SP) thymocytes migrate to the thymic medulla, where they undergo negative selection to eliminate autoreactive T cells and functional maturation to acquire immune competence and egress capability.

**Methodology/Principal Findings:**

To elucidate the genetic program underlying this process, we analyzed changes in gene expression in four subsets of mouse TCRαβ^+^CD4^+^CD8^-^ thymocytes (SP1 to SP4) representative of sequential stages in a previously defined differentiation program. A genetic signature of the migration of thymocytes was thus revealed. CCR7 and PlexinD1 are believed to be important for the medullary positioning of SP thymocytes. Intriguingly, their expression remains at low levels in the newly generated thymocytes, suggesting that the cortex-medulla migration may not occur until the SP2 stage. SP2 and SP3 cells gradually up-regulate transcripts involved in T cell functions and the Foxo1-KLF2-S1P_1_ axis, but a number of immune function-associated genes are not highly expressed until cells reach the SP4 stage. Consistent with their critical role in thymic emigration, the expression of S1P_1_ and CD62L are much enhanced in SP4 cells.

**Conclusions:**

These results support at the molecular level that single positive thymocytes undergo a differentiation program and further demonstrate that SP4 is the stage at which thymocytes acquire the immunocompetence and the capability of emigration from the thymus.

## Introduction

Acquisition of a complete and functional competent T cell repertoire requires that T cell progenitors undergo a tightly regulated developmental program. This eventful program includes T cell lineage commitment, V(D)J recombination at the γδ and αβ T cell receptor (TCR) loci, positive selection of T cells with appropriate MHC restriction, phenotypic proceeding of CD4^-^CD8^-^ (double-negative, DN) cells through CD4^+^CD8^+^ (double-positive, DP) to CD4^+^CD8^-^ or CD4^-^CD8^+^ (single positive, SP) cells, negative selection, and final maturation of SP cells [1], [2]. The latter two events occur in the thymic medulla whereas the rest take place in the thymic cortex.

For decades, SP thymocyte maturation has received the least attention and the cells are simply viewed as a nearly uniform population that has finished the positive selection and are waiting for the emigration from the thymus to the periphery. Recently, however, accumulating evidence suggested that SP thymocytes are heterogeneous and undergo an ordered differentiation program. The newly generated CD4^+^ and CD8^+^ SP cells gradually change their surface markers [3], [4], [5], acquire the resistance to apoptosis and capabilities to proliferate and produce cytokines when triggered with various stimuli [3], [6], [7], [8], [9]. Most importantly, T cells undergo negative selection to rid the repertoire of potentially autoreactive specificities [8], [10], [11]. After about 4–5 days of residence in the medulla, the proliferation competent “mature” CD4^+^ and CD8^+^ SP cells emigrate from the medulla and/or corticomedullary junction (CMJ) following homeostatic cues of the peripheral T cell pool [12], [13], [14], [15], [16]. In addition, some evidence suggests that the development of unique regulatory properties in subsets such as regulatory T (T_reg_) cells may take place in the medulla [17], [18].

Polarized migration from the cortex to the medulla, movement within the medulla, and normal medullary structure and components are essential for proper SP development, repertoire selection, and T cell function [19], [20], [21], [22]. Preventing SP cells from entering or accumulating in the medulla such as that in *Ccr7* ^-/-^ and *plt* mice, or defects in the medulla such as those caused by *Aire* and *Relb* deficiency could result in autoimmunity, strongly supporting the important role of the medullary microenvironment in SP thymocyte development, in particular, negative selection against self-reactivity [23], [24], [25], [26], [27], [28], [29], [30], [31], [32], [33], [34], [35], [36]. However, the molecular mechanisms govern the SP migration and development remain elusive.

We have previously resolved CD4^+^ SP thymocytes into four subsets: SP1 (6C10^+^CD69^+^), SP2 (6C10^-^CD69^+^), SP3 (CD69^-^Qa-2^-^), and SP4 (CD69^-^Qa-2^+^), of which the proliferation, cytokine production, and survival capacities showed a trend of SP1<SP2<SP3<SP4. Multiple approaches were used to demonstrate that these four subsets define a linear, multiple-stage developmental program for the newly generated CD4^+^ SP thymocytes prior to their exportation to the periphery [37]. A severe blockage at the transition from SP3 and SP4 was found in mice deficient in *Aire* and *Relb*, indicating that this transition could be an important checkpoint [37]. Further studies of the mechanisms governing this developmental program will require a systemic analysis of molecular changes in these SP subsets. To this end, the array-based and/or high throughput sequencing-based analysis will be extremely useful to investigate gene expression signatures. However, all the data currently available treat the SP thymocytes as a homogenous population and are not ready to be applied for the analysis of the genes involved in various aspects of the differentiation within SP thymocytes [38], [39], [40], [41], [42], [43], [44], [45], [46]. Thus, we performed here a comparative microarray analysis of these four subsets of TCRαβ^+^CD4^+^CD8^-^ thymocytes and found a clear signature of cell migration at the molecular level.

## Results

### Gene expression profile of single positive thymocytes

**Figure 1:**
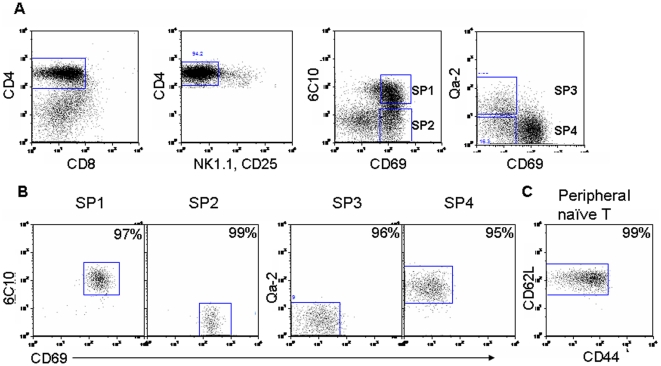
Isolation of CD4^+^ single positive thymocyte subsets and CD4^+^ naïve T cells from lymph nodes. (A–B) Thymocytes pooled from 15–20 mice were treated with anti-CD8 (3.155) mAb and complement. Viable cells were then separated by density centrifugation and stained with mAbs against mouse CD4, CD8 (53-6.7), CD69, and 6C10 or Qa-2. To exclude the contamination of NKT and Treg cells, PE-conjugated anti-NK1.1 and anti-CD25 antibodies were included in the mixture of staining Abs. CD4^+^CD8^-^CD25^-^NK1.1^-^ medullary thymocytes were gated (A) and sorted into the four subsets (B) defined in the text (i.e., SP1–SP4). The numbers in the top-right corners indicate the purity of the sorted cells. (C) Brachial, mesenteric, and inguinal lymph nodes were pooled from 5 to 8 mice and stained with mAbs against mouse CD4, CD8, CD44, CD62L, NK1.1, and CD25. CD4^+^CD8^-^CD44^lo/int^CD62L^hi^CD25^-^NK1.1^-^ naïve cells were sorted. The number indicates the purity of the sorted cells.

To identify genes involved in medullary thymocyte development, we compared genome-wide mRNA expression profiles of the four subsets of TCRαβ^+^CD4 SP thymocytes. We used antibodies specific for 6C10, CD69, and Qa-2 to separate these subsets. CD25^+^ and NK1.1^+^ cells were excluded to avoid the influence of mature and/or returning mature T_reg_ and NKT cells (Figure 1a). All populations were purified to 95–99% (Figure 1b), and total RNA was used to generate biotinylated cRNA. Complementary RNA from three independent preparations of SP1 to SP4 subsets was hybridized to Affymetrix murine gene arrays, containing 45101 probe sets. The data have been deposited in NCBI's Gene Expression Omnibus [47] and are accessible through GEO Series accession number GSE30083 (http://www.ncbi.nlm.nih.gov/geo/query/acc.cgi?acc=GSE30083).

**Figure 2:**
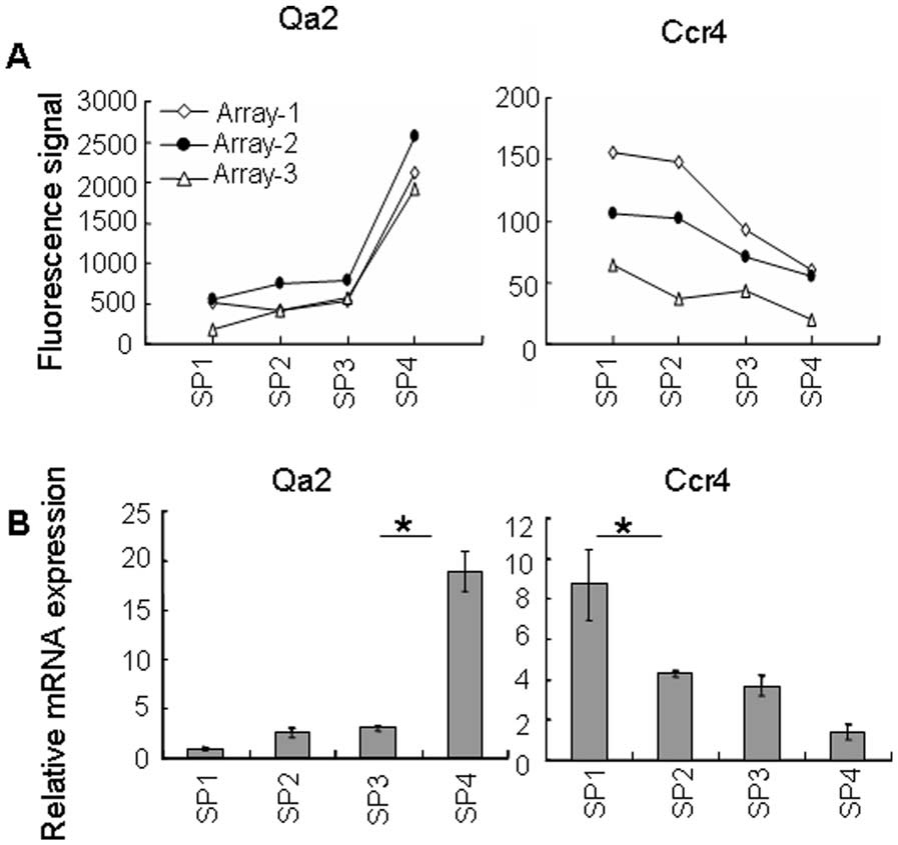
General validation of gene expression. (A) Gene expressions of Qa2 and Ccr4 in the four thymocyte subsets are revealed by the microarray analysis. (B) Quantitative RT-PCR results of Qa2 and Ccr4 transcripts in the four thymocyte subsets. Asterisks indicate significant (P<0.05) changes in each two-subset comparison. The experiment was performed for three times and similar results were obtained.

**Figure 3:**
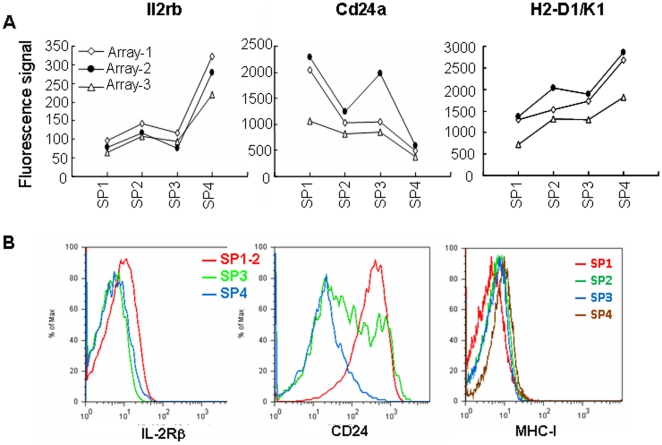
Expressions of Il2rb, Cd24, and H2-D1/K1 among the four subsets of CD4^+^ SP thymocytes. (A) Gene expression of Il2rb, Cd24a, and H2-D1/K1 by the microarray analysis. (B) Expression of IL-2Rβ, CD24, and MHC class I by flow cytometry.

To test the general validity of the chip-based hybridization, quantitative RT-PCR (qRT-PCR) and flow cytometry were used to confirm the differential expression of several genes among the four subpopulations. In addition, our initial cell purification strategy for the microarray analysis did not exclude a mature conventional T cell population (Qa-2^+^) that migrate from the periphery back to the thymus [48], [49], [50], [51], [52], the acquired array data in the SP4 subset may thus be contaminated by the presence of less than 5% of mature T cells (data not shown). Since most of these thymus-returning T cells have an activated/memory phenotype (CD44^hi^, composed of 80% of GFP negative CD4^+^ SP thymocytes in RAG2p-GFP transgenic mice, data not shown), the RNAs of the four SP subsets used in the qRT-PCR validation were differently sorted to exclude the NK1.1^+^, CD25^+^, and CD44^hi^ cells. Consistent with our cell purification strategy, CD25 and NK1.1 mRNAs were found to be expressed at extremely low levels both in arrays and in qRT-PCR (data not shown). Qa2 mRNA expression was largely restricted to SP4 cells in the array data and was confirmed by qRT-PCR (Figures 2a–b), agreeing well with the fact that Qa-2 was used as a marker to separate the SP4 cells. Other genes such as Ccr4, Cd24, Il2rb, and MHC class I also showed similar expression patterns in the array, qRT-PCR, and flow cytometry (Figures 2 and 3).

### Overview of the differentially expressed genes

**Figure 4:**
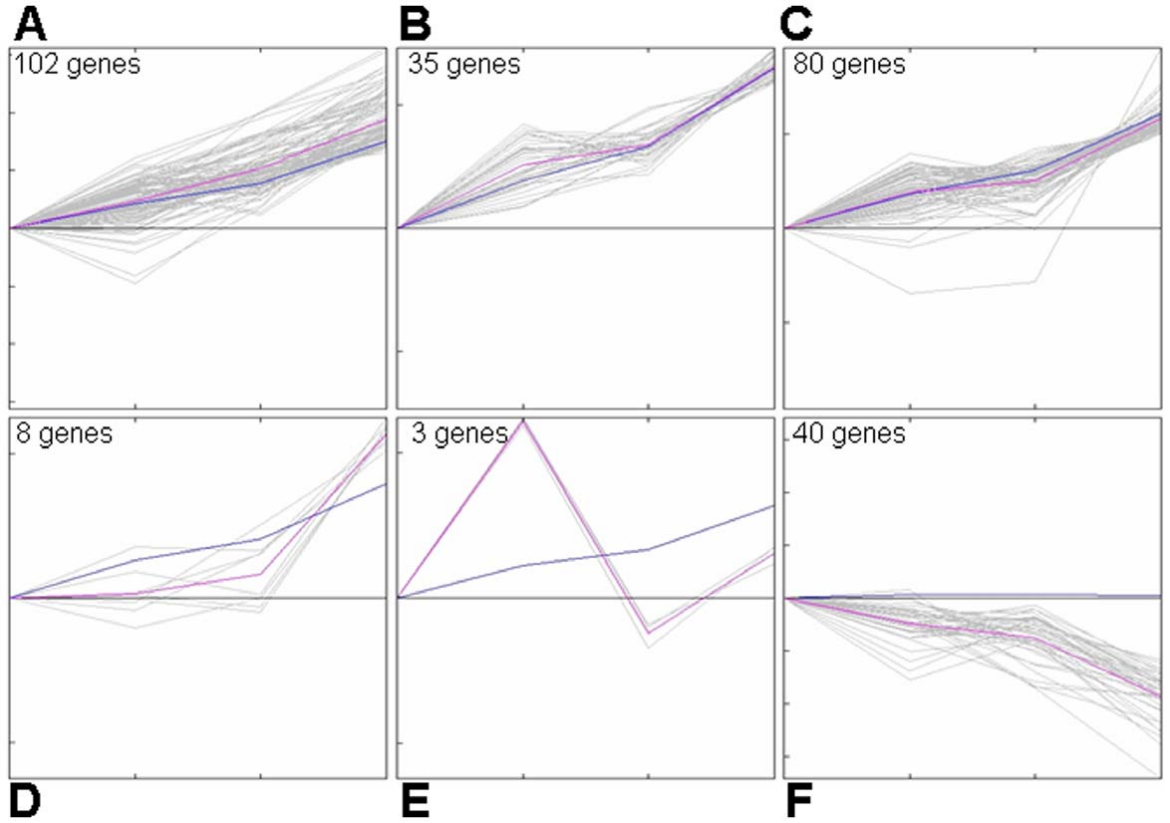
Patterns of expression kinetics among the four subsets of CD4 single positive thymocytes based on SOM analysis. (A–F) Six patterns of expression kinetics are shown.

Sets of probes whose expression increased or decreased by two-fold or more in any comparison were identified (see Table 1 for the number of genes and Table S1 for details). It is interesting to note that the majority of differentially expressed genes were enhanced along the developmental pathway (from SP1 to SP4). Based on the Kohonen's self-organizing maps (SOM analysis), six patterns of expression kinetics of the differentially expressed genes in the four subsets were further established. Roughly, they could be grouped into three categories: gradual increase (Figures 4a–d), gradual decrease (Figure 4f), and others (Figure 4e). Gene ontology (GO) analysis indicated that the genes within the gradual-increase category were related to such functions as immune response, lymphocyte activation, metabolic process, transcription, migration, and regulation of apoptosis. The KEGG pathway analysis revealed that cytokine-cytokine receptor (including chemokine-chemokine receptor) interaction, cell adhesion molecules, Jak-STAT signaling pathway, and immune response-related pathways were among the most significant pathways (Table S2). These data suggest that acquiring appropriate capabilities of migration, cytokine production, and immune function may be the major events in SP thymocyte development.

**Table 1:** Numbers of genes that are differentially expressed among the four subsets.

	Numbers of genes increased	Numbers of genes decreased
SP2 vs. SP1	4	0
SP3 vs. SP1	22	0
SP3 vs. SP2	20	20
SP4 vs. SP1	253	28
SP4 vs. SP2	160	14
SP4 vs. SP3	93	3

The numbers of genes that had greater than a 2-fold change in each two-subset comparison were separately listed as numbers of genes increased and decreased.

### Molecular signatures of functional maturation of SP thymocytes

**Figure 5:**
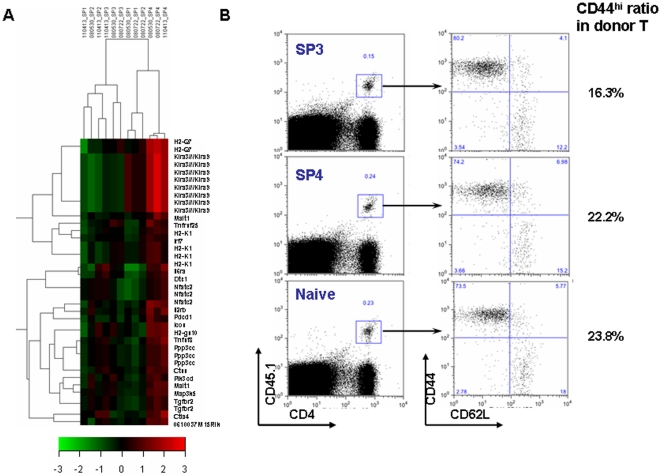
CD4 SP thymocytes gradually acquire immuno-competence. (A) Expression profiles of the immune-related genes among the four subsets. The profile was generated by hierarchical clustering using GeneCluster 2.0 package. All data from three array repeats were shown (080530_SPx, 080722_SPx, and 110413_SPx, x represents 1 to 4). Red represents up-regulation of gene expression, and green represents down regulation of gene expression. The entire list of genes is available in Table S2. (B) Similar activation of SP3, SP4 thymocytes and naïve CD4^+^ T cells upon Listeria Monocytogenes (Lm) infection. Purified SP3, SP4 thymocytes and naïve CD4^+^ T cells from C57BL/6 Ly5.1 congenic mice were adoptively transferred into C57BL/6 Ly5.2 mice (9×10^6^ per host mouse). One day later, the host mice were challenged with 2×10^4^ live Lm via tail vein injection. On day 7 of the infection, the cells from the lymph nodes and spleens were harvested and stained with CD45.1, CD4, CD44, and CD62L. The donor cells with the expression of both CD45.1 and CD4 are gated (shown in left paneal). The expression of CD44 and CD62L of donor cells (CD45.1^+^CD4^+^) are shown on the right panel.

By comparing various SP subsets directly purified from the thymus, we have shown that upon anti-CD3 and anti-CD28 stimulation *in vitro*, the SP1 and SP2 thymocytes undergo apoptosis and produce little or no cytokines whereas SP3 and SP4 cells have increased capabilities of proliferation and cytokine production [53]. This increase of functional competence along with the SP development was supported at the molecular level by the up-regulation of a variety of mRNAs including MHCs (Figures 3a–b), costimulatory molecules (Icos, Ctla4, Pdcd1, Tnfsf8), cytokines and their receptors (Il2rb, Il6ra, Tnfsf8, Tnfrsf25, Tgfbr2), and transcription factors and intracellular proteins that regulate immune responses (Pik3cd, Ppp3cc, Malt1, Ctss, Dtx1) (Figure 5a). Most of these significantly up-regulated molecules had highest expression in the cells at the SP4 stage, indicating the importance of this stage in the functional maturation of SP thymocytes. This gain of immune competence at the molecular level was further supported at the cellular level. When the same number of purified SP3, SP4 thymocytes and naïve CD4^+^ T cells from C57BL/6 Ly5.1 mice were adoptively transferred into congenic Ly5.2 mice, similar levels of donor T cell activation were observed seven days after Listeria monocytogenes infection of the host (Figure 5b). Surprisingly, unlike our previous finding that the SP3 cells proliferate less and produced less cytokines in vitro compared to the SP4 cells, we didn't see such significant differences in vivo. The functional maturation of the SP3 cells in the periphery, like that of the recent thymic emigrants (RTEs), may partially explain the differences between in vitro and in vivo [54], [55] (data not shown).

### Regulation of migration and adhesion during SP maturation

**Figure 6:**
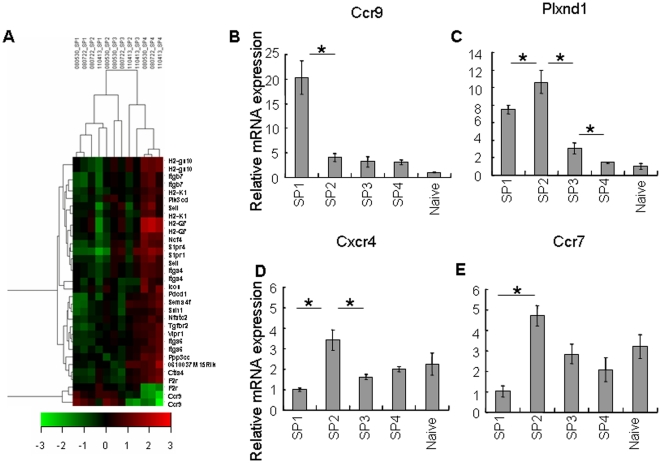
Molecular basis of the adhesion and migration of CD4 SP thymocytes during their final maturation process. (A) Expression profiles of the adhesion/migration-related genes among the four subsets. The profile was generated by hierarchical clustering using GeneCluster 2.0 package. All data from three array repeats were shown (080530_SPx, 080722_SPx, and 110413_SPx, x represents 1 to 4). Red represents up-regulation of gene expression, and green represents down regulation of gene expression. The entire list of genes is available in Table S2. (B–D) Gene expression of chemotactic molecules that have been reported to change after positive selection. Relative mRNA level of Plxnd1 (B), Ccr9 (C), Cxcr4 (D), and Ccr7 (E) in the four subsets of CD4 SP thymocytes as determined by qRT-PCR. Asterisks indicate significant (p<0.05) changes compared between the two subsets. Three experiments were performed on RNAs from three independently isolated cell populations for each thymocyte subset and similar results were seen.

The migration of SP thymocytes from the cortex to the medulla and the migration of SP thymocytes on medullary stroma are important for proper SP maturation and negative selection. The egress of SP cells from the thymus to the periphery also requires the cells capable of responding to the guidance from the periphery. Adhesion molecules and directional cues that are either attractive or repulsive may all contribute to the cell migration. As can be seen in our array data, there are a variety of changes in gene expression of chemokine receptors, adhesion molecules, cytokeletal molecules, signaling molecules, and other factors known to regulate cell motility in other systems (Figure 6a). The kinetics of these changes can be grouped into gradual increase and gradual decrease patterns as described previously. Interestingly, only one gene, Ccr9, fell into the continuously decreased expression profile. The decrease of Ccr9 mRNA level was confirmed by qRT-PCR in which the amount of Ccr9 transcript was significantly down-regulated in the SP2 cells and was lowest in the SP4 (Figure 6b). CCR9 is expressed in DP thymocytes and activated DP respond to CCR9 ligand (CCL25) expressed by cortical epithelial cells [34], [56]. The down-regulation of Ccr9 in SP2 thymocytes may relieve this subpopulation from retaining in the thymic cortex. PlexinD1 has been shown to suppress CCR9/CCL25 signaling and facilitate the medullary entry of positively selected CD69^+^ thymocytes [41]. Unlike the results from the array data where no significant differences were found, those from qRT-PCR showed high levels of Plxnd1 transcripts in SP1 and SP2 cells but significantly lower levels in SP3 and SP4 cells (Figure 6c). Compared to SP1 cells, the higher level of Plxnd1 in SP2 seems to correlate with the lower level of Ccr9 in these cells, facilitating their cortex-to-medulla migration. CCR7 is also an essential molecule in this type of migration [33], [38], [57]. Its deficiency leads to the accumulation of SP thymocytes in the cortex and the development of autoimmunity [33], [57]. Interestingly, the transcripts of Ccr7 were rather low in the newly generated SP thymocytes (SP1 cells), but were quickly up-regulated and maintained at high levels afterwards with the highest expression at the SP2 stage (Figure 6e). These expression patterns between SP1 and SP2 cells indicate that the cells at the SP2 stage might be the main population capable of migrating from the cortex to the medulla. The down-regulation of Ccr9 and up-regulation of Ccr7 and Plxnd1 may be coordinated to direct the SP2 thymocytes to enter the medulla. Other molecules that may contribute to the cortex-to-medulla migration include CCR4 and CXCR4. The expression of CCR4 in the thymocytes was reported to increase after positive selection while its ligands were produced predominantly in the medullary CD80^hi^ epithelial cells [58], [59], [60], [61]. CXCR4 also changes after positive selection but its role in the medullary entry is not very clear [62], [63], [64]. However, in our array and qRT-PCR results, the level of Ccr4 transcript was highest in the SP1 cells (a population that is likely derived from DPs after positive selection) and significantly down-regulated in the SP2 and other subgroups (Figures. 2a–b) whereas that of Cxcr4 showed a transient and mild (though significant) increase in the SP2 cells (Figure 6d). Thus, CCR4 and CXCR4 may not play a major role in directing the medullary entry.

**Figure 7:**
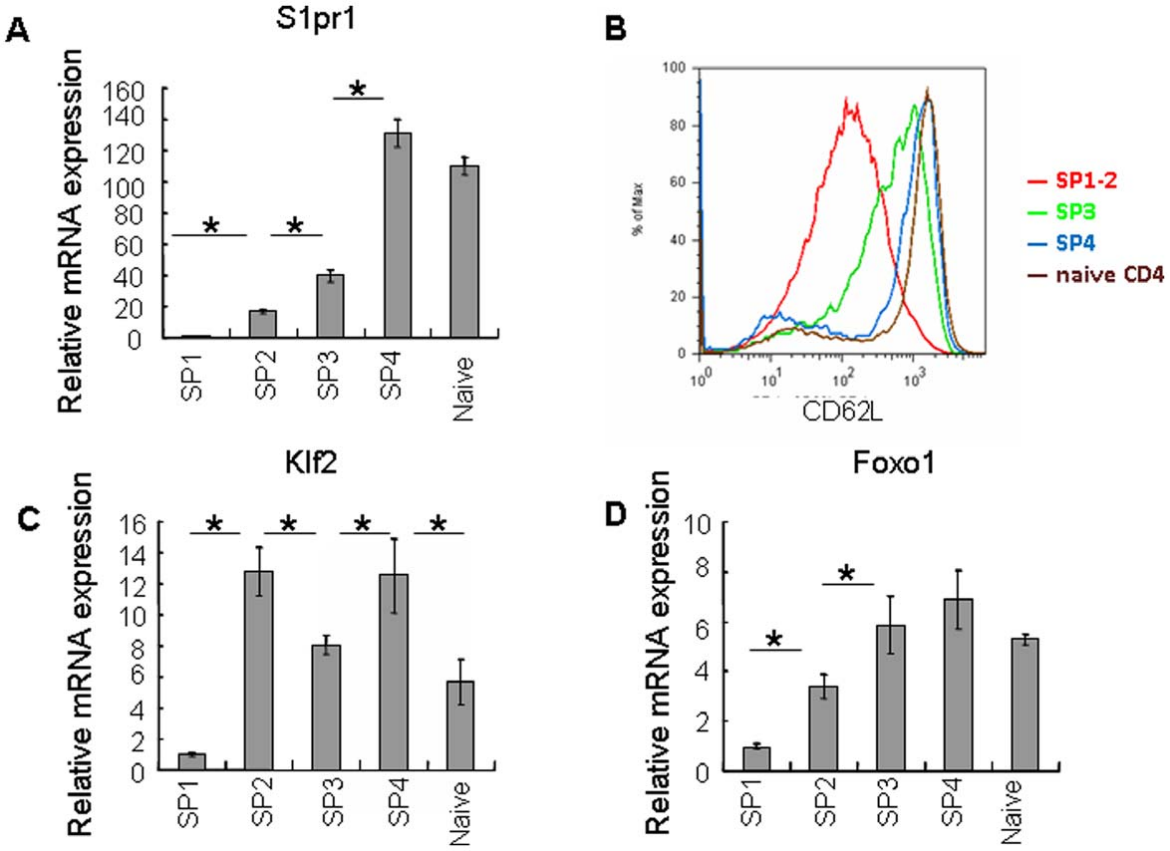
Expression of genes that are involved in the thymocyte migration and in Foxo1-Klf2 axis. (A, C–D) Relative mRNA amount of S1pr1, Klf2, and Foxo1 in naïve CD4^+^ T cells and the four subsets of CD4 SP thymocytes as determined by real time RT-PCR. (B) Expression of CD62L as determined by flow cytometry in peripheral naïve CD4^+^ T cells, double positive thymocytes, and the four subsets of CD4 single positive thymocytes. Asterisks indicate significant (p<0.05) change compared between the two subsets. The experiment was repeated for two to three times and similar results were seen.

**Figure 8:**
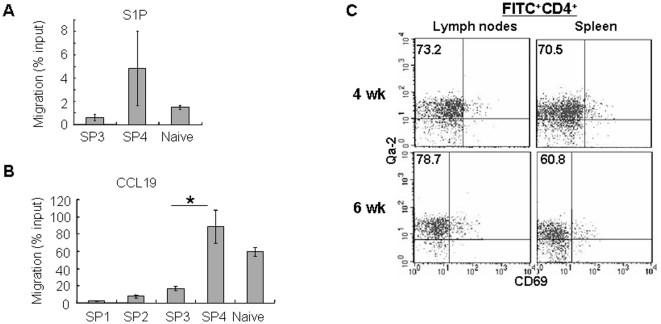
Cells with the SP4 phenotype are the main population in RTEs. (A-B) Transwell migration of SP thymocytes and naïve CD4^+^ T cells to S1P (A) and CCL19 (B). Three experiments were performed and the mean and standard deviation were shown. Asterisks indicate significant (P<0.05) difference between SP3 and SP4 subsets. (C) Phenotype of RTEs. Twenty-four hours after intrathymic injection of FITC, the cells from the lymph nodes and spleen were harvested and stained with CD4, Qa-2 and CD69. The surface expression of Qa-2 and CD69 in FITC^+^CD4^+^ T cells are shown. The numbers represent the percentage of Qa-2^+^CD69^-^ cells in FITC^+^CD4^+^ T cells. The experiment was repeated for two more times and similar results were obtained.

Eighteen genes demonstrated continuously increased kinetics in the microarray data. Among them, S1pr1 (S1P_1_) and Sell (CD62L) showed an up-regulation in the SP3 and SP4 subsets (data not shown). This trend of expression was confirmed by qRT-PCR of S1pr1 (Figure 7a) and FACS staining of CD62L (Figure 7b). It has been reported that both mature SP thymocytes and RTEs highly express S1P_1_ and CD62L [15], [21]. The surface receptor S1P_1_ allows SP thymocytes being exported from the thymus in response to S1P present in the circulation as well as produced by a thymic stromal cell type such as neural crest-derived perivascular cells [14], [15], [65], [66], [67]. CD62L enables the emigrants homing to the secondary lymphoid organs (SLO). We thus further examined the chemotaxis of SP thymocytes towards S1P. Consistent with the high expression of the receptor, S1pr1, in the SP4 subset, only cells from this group migrated towards S1P in a comparative way as naïve CD4^+^ T cells (Figure 8a). As a control of the migration assay, we chose CCL19 because the expression pattern of its receptor, CCR7, was different from that of S1pr1. The level of CCR7 transcripts was high in most of the CD4^+^ SPs except the cells at the SP1 stage (Figure 6e). Furthermore, CCR7 is essential in both cortex-to-medulla migration in the thymus and SLO homing in the periphery [68], [69]. Unexpectedly, however, the SP2 and SP3 cells did not respond to CCL19 as efficiently as the SP4 and naïve T cells (Figure 8b). Although the cause for this discrepancy is unclear, the results of both migration assays provide the functional evidence that the SP4 cells may be best equipped to leave the thymus. Indeed, when FITC was introduced into adult mice via intrathymic injection (a common approach to study RTEs), most of the FITC positive CD4^+^ T cells in the periphery (lymph nodes and spleen) had a Qa-2^+^CD69^-^ phenotype (SP4 phenotype) (Figure 8c). When purified SP1 thymocytes were injected into the thymus, we also found that most of the cells being exported had a SP4 phenotype [53]. In addition, studies with RAG2p-GFP transgenic mice by Fink group and ours consistently found that most of the GFP^hi^ T cells (representing RTEs) in the periphery of the adult mice have a Qa-2^+^ phenotype [54] (and data not shown). Together, these data suggest that the SP4 subset is the one in the thymus that acquires the capability to egress.

Kruppel Like Factor 2 (KLF2) is reported to directly regulate the expression of egress-enabling molecules S1P_1_ and CD62L [21], [70], [71]. Foxo1 has also been shown to be involved in regulating CD62L expression. Other molecules that are regulated by Foxo1 include KLF2 and CCR7. Mice deficient in Klf2 or Foxo1 were found to have similar failures in thymic egress as those lack S1P_1_ [68], [71], [72]. In our array data, both Klf2 and Foxo1 presented a gradually increased expression pattern from SP1 to SP4 cells whereas the qRT-PCR of Klf2 showed a significant (P<0.05) and sudden increase at SP2 (Figures 7c–d and data not shown). This significant (P<0.05) and abrupt up-regulation at the SP2 subpopulation was also seen in Ccr7 by both microarray and real time PCR (Figure 6e and data not shown). Interestingly, the transcripts of Sell and S1pr1 did not reach the highest levels until cells became SP4 (Figures 7a–b). Additionally, highest migratory capabilitiy towards CCL19, a CCR7 ligand, was not acquired until SP thymocytes reach the SP4 stage (Figure 8b). These unexpected late expressions in the SP4 subset suggest that additional events in the SP2 and/or SP3 stages may be required in the regulation of these molecules. In supporting this notion, SP3-to-SP4 transition was found to be blocked in Aire^-/-^ mice and whether the blockage affects the egress is currently under investigation [37], [61]. Taken together, these expression data strongly suggest that SP4 cells are preferentially exported from the thymus. The molecular program that regulates this migration may start from the SP2 stage and continue in the SP3 stage.

## Discussion

As many key events in T-lymphopoiesis including negative selection and T_reg_ lineage commitment are found to take place in the thymic medulla, the importance of single positive thymocyte differentiation and medullary microenvironment is gradually appreciated. However, the molecular basis that regulates this developmental program lacks a systemic and detailed analysis, rendering a much slower progress in the research in the SP compared to that in the DN and DP cells. In the present study, mRNA expression profiling by microarray was performed in the four subsets of SP thymocytes which have been previously shown to represent sequential stages in a defined differentiation program in the medulla. A clear molecular signature of cells acquiring functional competence was found. Molecules such as MHCs, costimulatory molecules, cytokines and their receptors showed a gradual increase in their expressions starting from SP2 or SP3 subpopulation, in concert with our previous finding that the development of SP1 to SP4 follows a progressive phenotypic and functional maturation process [37], [53].

During this maturation process, a transition from SP3 to SP4 was found to be critical as a blockage of this transition in *Aire* ^-/-^ and *Relb* ^-/-^ mice is associated with high incidence of autoimmune diseases. Whether this blockage mainly affects the deletion of autoreactive T cells or affects the thymocyte maturation as well is not clear. In our array data, there is a sharp increase of multiple gene transcripts in the SP4 cells. Considering that the normality of thymic stromal cells is required for the SP3 to progress to SP4 [37], this type of abrupt and significant up-regulation of functional genes in the SP4 cells may be partially driven by the signals from medullary stromal cells. The expressional changes of which molecules require such a thymocyte-stromal cell interaction and/or facilitate the transition of SP3 to SP4 cells are currently under investigation. Another possibility of the significant expressional changes in the SP4 cells may be derived from our purification strategy and the impurity of SP4 cells. Although we made an effort to exclude T_reg_, NKT, and thymus-reentered activated T cells (CD25^+^, NK1.1^+^, and CD44^hi^) from the SP4 subset, SP4 thymocytes may still be heterogeneous, containing cells that are on their way to become mature T_reg_ and NKT and trace amount of naïve T cells that migrate back to the thymus. The molecular signature but not necessarily the cellular function may be affected by this type of contamination. The use of RAG2p-GFP transgenic mice and/or MHC class II-restricted RAG knockout TCR transgenic mice may help to obtain more homogeneous cell population.

The orchestrated migration of developing thymocytes through various specialized microenvironments in the cortex is essential for proper T cell development. But less is known of the thymocyte trafficking from the cortex to the medulla and within the medullary environment. Similarly, although the widely accepted mechanism for thymocytes' exiting the thymus is S1P_1_-mediated migration, when do SP cells express S1P_1_ and how to prevent the premature exit of autoreactive cells at the cortico-medullary junction is still not clear [73]. Our array and qRT-PCR results on the four SP subsets suggest a route by which the newly generated SP thymocytes migrate within the thymus: the SP2 cells down-regulate CCR9, up-regulate CCR7 and plexinD1, and migrate from the cortex to the medulla; the SP2 and SP3 cells gradually up-regulate molecules involved in T cell functions and the Foxo1-KLF2-S1P_1_ axis; the high expressions of S1P_1_, CCR7, and CD62L in SP4 cells enable these functional competent cells to leave the medulla and enter the periphery. These results provide fine evidence to support the previous finding that the emigration of SP thymocytes is not random and only a subpopulation of SP cells that finish the series of maturational events are capable of exiting the thymus [16]. Development of self tolerant and fully matured SP cells rely heavily on this ordered trafficking as defects in SP thymocyte migration from the cortex to the medulla, such as *Ccr7* deficiency, resulted in the ectopic development of SP in the cortex/cortex-medullary junction and autoimmunity in the periphery. Similarly, defects in the thymocytes-epithelial cells interaction in the medulla, such as *Aire* and *Relb* deficiency, result in the SP3-to-SP4 blockage and autoimmunity in the periphery [37], [53].

It remains unknown how this highly ordered process is regulated at the molecular level and whether positive selection in the thymic cortex alone or together with signals from the medullary microenvironment including negative selection drive these events to occur in a sequential order. A study of Egr1-deficient mice suggests that positive selection could have an impact on the survival of cells further down the thymocyte developmental pathway and even after entering the periphery [74]. Foxo1 and the Foxo1-KLF2-S1P_1_ axis could be affected in mice with defects in positive selection due to the fact that the expression and activity of Foxo1 requires TCR stimulation and PI3K [75]. Medullary stromal cells, however, also likely play an important role in SP development in addition to the elimination of autoreactive T cells. At least four pieces of evidence in our data support this, 1) the high expression of immune function-associated genes was mostly seen in the SP4 subset (Figure 5a); 2) although Foxo1 and Klf2 were up-regulated in the SP2 cells, their downstream genes, S1pr1 and Sell, were not fully expressed until cells reached the SP4 stage (Figures 7a-b); 3) only SP4 cells showed a comparative response to CCL19 as naïve T cells despite of an early upregulation of Ccr7 mRNA level in the SP2 subset; 4) the transition of cells from the SP3 to SP4 likely requires a normal medullary microenvironment as defects in the stromal cells (such as those in *Aire* ^-/-^ mice) could block SP3-to-SP4 transition [37], [53]. Thus, SP2 and especially SP3 may be key stages at which thymocytes receive signals from medullary environment including stromal cells.

Taken together, this is the first detailed analysis of the gene expression profiles of CD4 SP subsets. It reveals at a molecular level a clear map of how CD4 SP thymocytes gradually acquire the functionally matured, and ready-to-egress properties. It also provides transcriptomic evidence supporting the model of “maturation-dependent emigration” of SP thymocytes [16]. It established a molecular basis to further study the signals that initiate and govern these progressive changes and to understand what signals are obtained from the positive selection and what from the medullary microenvironment.

## Materials and Methods

### Mice and Ethics Statement

Congenic C57BL/6 Ly-5,1 (CD45.1) and C57BL/6 Ly-5.2 (CD45.2) mice between 6-8 weeks of age were used throughout this study. The animals were kept in a specific pathogen-free facility at Peking University Health Science Center (Beijing, China). The experimental procedures on use and care of animals had been approved by the ethics committee of Peking University Health Science Center with an approval number of bjmu20110301.

### Antibodies

Anti-CD8 (3.155) was prepared from hybridomas obtained from the American Type Culture Collection. The 6C10 (SM6C10) was from Dr. Linna Ding (National Institutes of Health) and was subsequently labeled with FITC. Antibodies against CD4 (H129.19 or RM4-5), CD8 (53-6.7), TCRβ (H57–597), CD69 (H1.2.F3), Qa-2 (H1-1-2), NK1.1 (PK136), CD44 (IM7), CD62L (MEL-14), CD24 (M1/69), IL-2Rβ (5H4), streptavidin-PE/APC, and isotype control antibodies were purchased from BD Pharmingen. Anti-Qa-2 (H1-1-2) and CD25 (PC61) were from eBioscience.

### Isolation of peripheral naïve CD4^+^ T cells and subsets of CD4 SP thymocytes

Single-cell suspensions of thymocytes derived from C57BL/6 mice at 6-8 weeks of age were treated with anti-CD8 (3.155) mAb and complement (guinea pig sera) to remove CD8^+^ cells. After two cycles of killing and removal of dead cells by density centrifugation, the viable cells were stained with CD4-PerCP-Cy7, CD8-APC-Cy7 (53-6.7), CD69-PerCP-Cy5.5, 6C10-FITC, Qa-2-Biotin-strepavidin-APC, CD25-PE, and NK1.1-PE and then subjected to cell sorting (FACSAria). For the RNAs used in qRT-PCR validation, the cells were further stained with CD44-PE to purify the CD44^lo^ cells. Four subsets of CD4^+^CD8^-^ thymocytes were thus obtained: SP1 (6C10^+^CD69^+^Qa2^-^), SP2 (6C10^-^CD69^+^Qa2^-^), SP3 (6C10^-^CD69^-^Qa2^-^), and SP4 (6C10^-^CD69^-^Qa2^+^). The naïve CD4^+^ T cells (CD4^+^CD44^lo/int^CD62L^hi^CD25^-^NK1.1^-^) were purified from the lymph node cells from the same mice. The purity of individually sorted groups was 95–99% as reanalyzed on FACS (Fig. 1a–b). The expression of TCRβ was checked among these purified cell subsets and 99.9% of the cells were TCRβ^+^ (data not shown).

### Microarray analysis

Total RNA was isolated from each SP subset using Trizol (Invitrogen) according to the manufacturer's instructions. RNA concentrations were determined by spectrophotometry. Only those samples that showed no degradation (ratios approaching 2∶1 for the 28S and 18S bands) in formaldehyde agarose gel electrophoresis were used to generate labeled targets. The RNA samples were then sent to Bioassay Laboratory of CapitalBio Corporation (Beijing, China) for amplification, biotin labeling, hybridization to Affymetrix Mouse Genome 430 2.0 Arrays (Affymetrix). Three arrays were used for each SP subsets. The hybridization data were analyzed using Affymetrix® GeneChip® Operating Software Version 1.4 (GCOS 1.4). The scanned images were first assessed by visual inspection and analyzed to generate raw data files saved as CEL files using the default setting of GCOS 1.4. A Robust Multi-array Analysis normalization procedure was performed to normalize the different arrays using R bioconductor. The noise and 3′ to 5′ signal ratios of all arrays were listed in Table 2. The data have been deposited in NCBI's Gene Expression Omnibus [47] and are accessible through GEO Series accession number GSE30083 (http://www.ncbi.nlm.nih.gov/geo/query/acc.cgi?acc=GSE30083).

**Table 2:** Quality control data from 16 arrays.

	Noise (Mean±STDEV)			Gapdh 3′/5′ signal ratios		
	Repeat 1	Repeat 2	Repeat 3	Repeat 1	Repeat 2	Repeat 3
SP1	1.21±0.04	0.9±0.03	1.16±0.03	1.01	1.00	1.10
SP2	1.13±0.05	0.89±0.02	1.13±0.04	1.05	1.04	1.08
SP3	1.16±0.05	1.00±0.04	1.07±0.02	1.03	0.99	1.17
SP4	1.12±0.04	0.84±0.03	1.12±0.02	1.01	1.07	1.14

In the comparison analysis, a two-class unpaired method in the Significant Analysis of Microarray software (SAM) was applied to identify differentially expressed genes between test and control groups. The selection threshold of false discovery rate is FDR<5% and fold change>2.0 in the SAM output result.

Clustering of self-organizing maps (SOM) was performed by GeneCluster 2.0 package and 9 class (3×3) SOMs were constructed. To organize genes into hierarchical categories and uncover gene regulatory network on the basis of biological process and molecular function, the differentially expressed genes were mapped to Gene Ontology (GO) terms and Kyoto Encyclopedia of Genes and Genomes (KEGG) pathways using MAS (molecule annotation system, http://bioinfo.capitalbio.com/mas) platform. GO terms and KEGG pathways with false discovery rate (FDR)-corrected P values <0.05 were considered statistically significant.

### Quantitative RT-PCR

RNA was purified from isolated thymocytes using TRIZOL (Invitrogen) and was used to make cDNA using random primers and the Reverse Transcription System (Promega). For quantitative Real-Time PCR, iQ™ SYBR® Green Supermix (Bio-Rad) was used according to the manufacturer's instructions. Quantitative PCR was performed on an iCycler (Bio-Rad Ltd, Hemel Hempstead, England, UK), with each sample in triplicate, at an RNA equivalence of 15 ng/well. Oligos for each gene are listed in Table 3. PCRs were performed for 40 cycles at 95°C for 30 s, 58°C for 30 s, and 72°C for 20 s. The quantification was based on ΔΔCT calculations and were normalized to GAPDH as loading controls and calibrated to SP1 levels in Figures 2b left, 6d–e, and 7a, c–d; to SP4 levels in Figure. 2b right; and to naïve T cell levels in Figures 6b–c. RT-PCR analysis was carried out on RNA from three independently isolated cell populations for each thymocyte subset and naïve T cells. Statistical analysis of the results obtained from quantitative RT-PCR was performed using GraphPad Prism 5 software (San Diego, CA, USA). Student's *t*-test was used to evaluate the significance of the differences between group means. Statistical significance was defined as *P*<0.05.

**Table 3:** Primers for selected genes analyzed using real-time PCR.

	Forward (5′ - 3′)	Reverse (5′ – 3′)
GAPDH	CGGCCGCATCTTCTTGTGCA	GCAAATGGCAGCCCTGGTGAC
Qa2	CAGGTCTTATGGTGCTGTCA	GCATGTGTAATTCTGCTCCTTC
Ccr4	GGAAGGTATCAAGGCATTTGGG	GTACACGTCCGTCATGGACTT
S1pr1	TCGCCGACAGCAGCAAGATG	AAAGCCAGGTCAGCGAGCAATC
Klf2	CTCAGCGAGCCTATCTTGCC	CACGTTGTTTAGGTCCTCATCC
Foxo1	AGGGCGACAGCAACAGCTCG	TCCATGGACGCAGCTCTTCTCC
Ccr7	GCACCATGGACCCAGGGAAACC	TGAGAGGCAGGAACCAGGCCT
Plxnd1	TGCAGGCTGCCATTCGTGCA	GGCCGCTCCGCAATCCAGTT
Ccr9	CTTCAGCTATGACTCCACTGC	CAAGGTGCCCACAATGAACA
Cxcr4	AGGCGTTTGGTGCTCCGGTA	TTCATCCCGGAAGCAGGGTTCCT

### Flow cytometry

Single-cell suspensions of thymocytes from C57BL/6 mice were removed of CD8^+^ cells and stained with CD4-PerCP-Cy7, CD8-APC-Cy7 (53-6.7), CD69-PerCP-Cy5.5, Qa-2-Biotin-strepavidin-APC, CD25-PE, CD44-PE, and NK1.1-PE. The expressions of CD62L, CD24, and IL-2Rβ among three subsets of CD4^+^CD8^-^ cells, including SP1-2 (CD69^+^Qa2^-^), SP3 (6C10^-^CD69^-^Qa2^-^), and SP4 (6C10^-^CD69^-^Qa2^+^), were obtained. The lymph node cells from the same mice were stained with CD4-PerCP-Cy7, CD8-APC-Cy7 (53-6.7), CD44-APC, CD62L-FITC, CD25-PE, and NK1.1-PE and the CD62L level of the naïve CD4^+^ T cells (CD4^+^CD44^lo/int^CD25^-^NK1.1^-^) was obtained and compared with that of the thymocyte subsets.

### RTE analysis

Mice were anesthetized with 1% napental via intraperitoneal injection. An incision was made in the sternum to reveal the thymus, and approximately 10–20 µl of FITC solution (1 mg/ml) was injected into each thymic lobe with a 30-gauge needle. Control mice were injected with PBS. The chest was closed with surgical clips in the overlying skin. FITC positive cells from the spleen and lymph nodes were analyzed 24 hours later.

### Listeria infection

SP3, SP4 thymocytes and naïve CD4^+^ T cells from C57BL/6 Ly5.1 congenic mice were purified by flow cytometry and were adoptively transferred into C57BL/6 Ly5.2 mice at 6–8 weeks of age (9×10^6^ per host mouse). Three mice in each group. One day later, the host mice were challenged with 2×10^4^ live Listeria Monocytogenes (a kind gift from Prof. Qibin Leng, Laboratory of Immune Regulation, Institute Pasteur of Shanghai, Chinese Academy of Sciences) via tail vein injection. On day 7 of the infection, the mice were sacrificed and the cells from the lymph nodes and spleens were stained with CD45.1, CD4, CD44, and CD62L. The donor cells with the expression of both CD45.1 and CD4 were gated.

### Transwell migration

Thymocyte subpopulations and naïve CD4^+^ T cells from C57BL/6 mice were purified and rested in migration medium at room temperature for one hour. The cells were then used for three-hour migration assays through Transwells (5 mm; Corning). S1P (Sigma) was used at 50 nM; CCL19 (R&D Systems) was used at 250 ng/ml.

## Supporting Information

Table S1 **Differentially expressed genes among the four subsets of CD4 SP thymocytes.** Sets of probes whose expression increased (red) or decreased (green) by two-fold or more in any comparison are listed.
(XLS)

Table S2 **KEGG pathway analysis of the differentially expressed genes using MAS platform.** The false discovery rate (FDR)-corrected P values <0.05 are considered statistically significant.
(XLS)
